# Accelerating Flux Calculations Using Sparse Sampling †

**DOI:** 10.3390/mi9110550

**Published:** 2018-10-26

**Authors:** Lukas Gnam, Paul Manstetten, Andreas Hössinger, Siegfried Selberherr, Josef Weinbub

**Affiliations:** 1 Christian Doppler Laboratory for High Performance TCAD, Institute for Microelectronics, TU Wien, Vienna 1040, Austria; manstetten@iue.tuwien.ac.at (P.M.); weinbub@iue.tuwien.ac.at (J.W.); 2 Silvaco Europe Ltd., St. Ives PE27 5JL, UK; andreas.hoessinger@silvaco.com; 3 Institute for Microelectronics, TU Wien, Vienna 1040, Austria; selberherr@tuwien.ac.at

**Keywords:** flux calculation, etching simulation, process simulation, topography simulation

## Abstract

The ongoing miniaturization in electronics poses various challenges in the designing of modern devices and also in the development and optimization of the corresponding fabrication processes. Computer simulations offer a cost- and time-saving possibility to investigate and optimize these fabrication processes. However, modern device designs require complex three-dimensional shapes, which significantly increases the computational complexity. For instance, in high-resolution topography simulations of etching and deposition, the evaluation of the particle flux on the substrate surface has to be re-evaluated in each timestep. This re-evaluation dominates the overall runtime of a simulation. To overcome this bottleneck, we introduce a method to enhance the performance of the re-evaluation step by calculating the particle flux only on a subset of the surface elements. This subset is selected using an advanced multi-material iterative partitioning scheme, taking local flux differences as well as geometrical variations into account. We show the applicability of our approach using an etching simulation of a dielectric layer embedded in a multi-material stack. We obtain speedups ranging from 1.8 to 8.0, with surface deviations being below two grid cells (0.6–3% of the size of the etched feature) for all tested configurations, both underlining the feasibility of our approach.

## 1. Introduction

**Figure 1:**
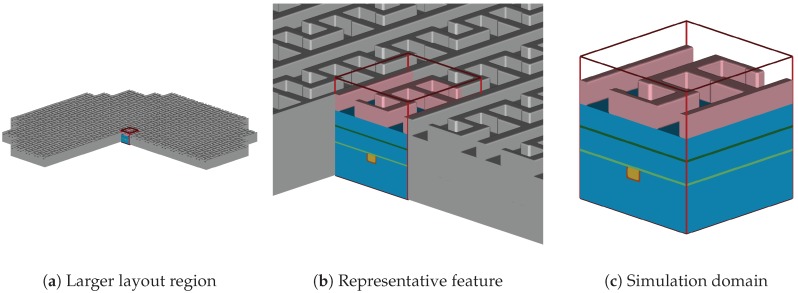
(**a**) a larger region of a layout on a wafer; (**b**) close-up on a representative feature; (**c**) feature-scale simulation domain with domain boundaries in red.

Semiconductor process simulations can be partitioned into two major types: reactor-scale and feature-scale simulations. The former simulates the full reactor chamber whereas the simulation domain of the latter is a small region of the wafer surface (see Figure 1). Feature-scale simulations (which are the focus of this work) are used when the detailed topographical behavior and the prediction of the surface evolution is of major interest, instead of focusing on the global behavior of the wafer in a reactor-scale simulation [1].

Particularly challenging is the simulation of etching processes, where the large number of possible process parameters and available materials renders conventional evaluation unreasonable considering costs and time [2], since even the smallest change in an experimental setup can have a major impact on the properties of a device [3]. Additionally, the more and more used three-dimensional (3D) designs (e.g., FinFET [4] or 3D NAND flash memory [5]) prohibits the problem reduction to two dimensions as a simple yet effective technique to minimize computational effort for larger 3D structures. Hence, the development of faster algorithms and methods is a substantial objective to solve the increasingly complex 3D problems and thereby sustaining the high pace of ultra-large scale integrated circuit developments [2].

The common computational tasks in a single timestep of a feature-scale etching simulation are (a) the preparation of a suitable surface representation for the surface flux rate calculations, (b) the calculation of the surface flux rate distributions on the surface, (c) the evaluation of the surface velocity models using the surface flux rate distributions, and (d) the advection of the surface according to the computed surface velocity field. Typically, the majority of the computational time is spent on the calculation of the flux rates [6,7]. In turn, the models for the etch rates depend on the flux rates of the involved etchants where the flux rate calculations constitute the main computational bottleneck.

The common flux calculation methods (which assume ballistic particle transport) rely on a large number of ray-surface intersection tests. Most frameworks are based on an implicit representation of the surface, using the level-set method for advection [8,9,10]. In [11], the authors study the Bosch process for a micro-electromechanical system (MEMS) application with Silvaco’s Victory Process simulator, which uses an implicit representation of the surface during ray casting [12]. However, during ray casting, different (temporary) representations for the surface are also used. Ertl et al. use the ViennaTS [13] simulator to investigate the Bosch process [14]. The process is simulated in three dimensions and the flux calculation is based on a Monte Carlo approach. A representation of the surface as a set of overlapping disks is used to approximate a closed surface. Heitzinger et al. showed a method to accelerate such Monte Carlo based flux calculations by coarsening the explicit surface mesh representing the surface [15]. Recently, Yu et al. presented a 3D topography simulation of a deep reactive ion etching (DRIE) process [16]. Therein, the surface is approximated using a set of spheres during the Monte Carlo ray tracing task.

A common way to increase the computational performance of ray casting is the use of hierarchical spatial acceleration structures, where a bounding volume hierarchy (BVH) is a common choice [17,18]. A similar approach is to apply a bi-level spatial subdivision for reducing the search space for an explicit surface representation in order to accelerate the Monte Carlo based ray tracing as shown by Yu et al. [19]. This method relies on a proper subdivision tree and an optimal choice of the underlying parameters, which need to be determined beforehand. Naeimi et al. accelerate their ray-tracing based heat transfer model by merging empty voxels, explicitly representing their geometry, inside their static simulation domain [20]. Hence, when a ray is traversing through the domain, it can quickly traverse the empty voxels, since they are much bigger than those on the respective surface. Although their algorithm provides a considerable speedup concerning the underlying ray-tracing, the voxel merging process can have a major impact on the performance in transient simulations, when it has to be applied in each timestep. Aguerre et al. proposed a method to reduce the computational effort if the flux calculation is radiosity based [21]. The method relies on a hierarchical strategy and a sorting scheme for acceleration of the necessary calculations based on explicit meshes. Bailey showed that an adaptive sampling of the geometrical elements can improve the computational performance of ray tracing tasks [7]. In [22], it is shown that the overhead introduced by generating a temporary explicit mesh in each timestep is by far compensated using optimized computer graphics libraries for single-precision ray casting.

Another acceleration approach was presented in [6], where the computational performance of the flux calculation is improved by evaluating the surface speed only on a sparse set of cells on an explicit surface mesh. This set is found using an iterative partitioning of the surface, without adapting the surface mesh itself. The approach is evaluated using a simple 3D single-material etching simulation of a cylindric hole. The computational performance of the flux calculation is increased by a factor of 2–8 (compared to the conventional evaluation on the mesh using all cells) while preserving the geometrical features of the surface.

In this paper, we extend the method presented in [6] by including multi-material interfaces into the partitioning scheme and by evaluating practically relevant multi-material problems. We evaluate the new scheme by simulating the etching of a dielectric layer as part of a Dual-Damascene fabrication process sequence, which is an important technique in today’s semiconductor industry [23,24]. We show that the novel flux calculation based on the sparse set of surface cells significantly improves the computational performance for practically relevant multi-material simulations, achieving speedups of 1.9 to 8, which is in the range of the speedups reported in [6]. Additionally, we show that the resulting surface deviations for the sparse set are below two grid cells after many timesteps (corresponding to 0.6 to 3% of the via size) for a wide range of surface resolutions. The study is completed with an in-depth analysis of the parallel performance of the sparse and dense flux calculation approaches.

## 2. Materials and Methods

### 2.1. Dual-Damascene Process

To reduce the required processing steps in the fabrication pipeline, the Dual-Damascene process was introduced in the 1990s and became an industry standard [25]. In this process, only a single metal deposition step is required, where vias and trenches are filled simultaneously, typically using copper as filling metal [26]. Additionally, the number of chemical-mechanical planarization steps as well as the number of deposition steps for the dielectric are reduced. This yields a shorter overall processing time together with less possibilities for failures.

In general, there are three different major Dual-Damascene process sequences: (1) trench-first, (2) via-first, and (3) self-aligned or buried-via. In the trench-first sequence, the etching of the trenches is conducted before the etching of the vias, whereas, in the via-first approach, the etching is done vice versa. Both methods apply a metal deposition step after each etching step, where the via and the trench are filled with copper. The self-aligned Dual-Damascene process etches both the via and the trench at the same time [25,27] and is the target application of this work.

**Figure 2:**
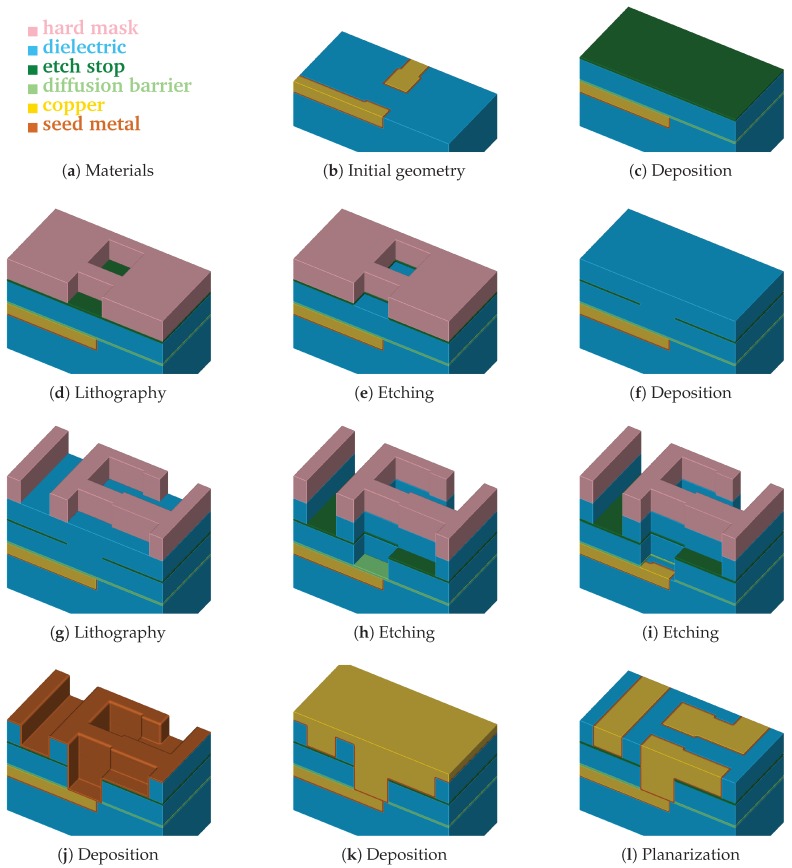
Individual processing steps for the self-aligned Dual-Damascene process. The material legend is shown in (**a**). (**b**–**l**) illustrate all processing steps to create a metalization layer on top of an exiting metalization layer (the square domain is clipped along one axis for better visibility). First row (**b**,**c**): initial patterned planar conductor and deposition of three layers: a diffusion barrier, a dielectric material, and an etch stop material. Second row (**d**–**f**): patterning of the etch stop layer (at the position of the vias) including the deposition of the second dielectric layer. Third row (**g**–**i**): patterning of the dielectric layers (vias and lines) including opening of the diffusion barrier layer around at the position of the via. Last row (**j**–**l**): copper metalization including the deposition of a metal seed layer and chemical-mechanical planarization after the copper deposition.

Figure 2 depicts a typical sequence of processing steps in the self-aligned Dual-Damascene process. The different steps show the initial surface of the wafer (Figure 2b), which is followed by the deposition of the diffusion barrier, a dielectric, and the etch stop (Figure 2c). Afterwards, a hard mask is deposited and subsequently the etch stop layer is patterned (Figure 2d,e). After the removal of the hard mask, a second dielectric is deposited and a hard mask is patterned again (Figure 2f,g). The next step is the combined etching of trenches and vias (Figure 2h). Afterwards, the finalizing steps are the removal of the etch barrier (Figure 2i), the deposition of the metal seed layer (Figure 2j), the copper deposition (Figure 2k), and the chemical-mechanical planarization (Figure 2l).

After switching from Aluminum to Copper as interconnect material in the early 2000s to overcome the problems surfacing in the sub-micron regime [25], today, new challenges arise on the continued path of miniaturization down to a few nanometers. For example, to reduce the interconnect capacitance, new dielectrics such as SiO_2_ or porous SiCOH have to be used [28]. As previously discussed, computer simulations provide a highly attractive option to assess the different etching behaviors of various materials to determine process designs and optimizations before proceeding with actual, conventional experimental investigations. Therefore, within this work, we show the applicability of the approach presented in [6] using the etching process of a dielectric layer (Figure 2g–h) as a practically relevant example of a multi-material simulation.

### 2.2. Multi-Material Simulation Framework

A single-material simulation framework [6] was developed to investigate efficient flux calculation methods for 3D etching and deposition processes. It is based on the sparse volume data structure of OpenVDB [29,30] and uses the accompanying tools to handle surface advection and surface extraction. All advection steps described in the following are performed using the level-set representation of the surface. The explicit representation of the surface, which is only used during the flux calculation, is obtained using OpenVDB’s “volumeToMesh” routine producing quads. We subdivide each potentially non-planar quad into two triangles using the distance to the zero-level-set as guidance for the subdivision pattern. The ray-surface intersection tests are performed using Embree [31]. In the following, we provide a short summary of the method to set the stage for the subsequent discussion on the improved multi-material iterative partitioning scheme and the detailed analysis of plasma etching steps for a Dual-Damascene process.

In each timestep of the simulation, the main computational tasks are (a) the calculation of the flux rates *R* on the surface, (b) the evaluation of the normal surface velocity $V_{n}$ (which depends on the flux rates), and (c) the advection of the surface. Typically, the normal surface velocity $V_{n}$ during an etching process depends on the flux rates *R* of the involved particle species

(1) $$V_{n}\left(x\right)=f\left(R_{1},R_{2},\ldots ,R_{k}\right),$$

where *k* denotes the number of different particle species. The flux rates depend on the incoming flux distribution $\Gamma_{in}$. In the simplest case, all incoming directions are equally weighted, which results in

(2) $$R\left(x\right)=\int_{\Omega }\Gamma_{in}\left(x,\omega_{d\Omega }\right)d\Omega ,$$

with ω being the incoming direction, and Ω denoting the upper hemisphere facing the source plane.

The surface of this hemisphere is discretized using a subdivided icosahedron. The directions from a surface point towards the centroids of the triangular discretization of the hemisphere are tested for visibility of the source. After obtaining the visibility information for all directions, a numerical integration is performed over the visible solid angles using a centroid rule [22].

**Figure 3:**
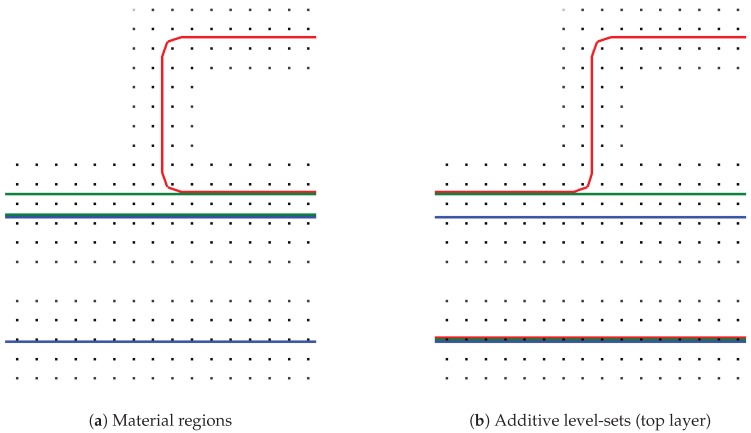
Three materials stack with a thick bottom region (blue), a thin layer (green), and a mask (red). (**a**) cross section of the material regions showing the level-set grid points and the extracted level-sets; (**b**) additive level-sets scheme from bottom to top (blue, green, and red).

Typically, different material regions are present during an etching process simulation, e.g., a dielectric patterned with a hard mask (Figure 2g). A straightforward approach would be to represent each material region with a corresponding level-set function, as shown in Figure 3a. To simultaneously advect all material regions, each region would then be advected separately, leading to potentially mutual penetration. In this case, the parts of a region which are penetrated by another region would be treated inactive, i.e., not subject to advection. One approach would be to perform Boolean operations between material regions to dissolve the penetrations. Hence, a strategy to decide which material fills the former penetrated volume is needed.

We extended the simulation framework presented in [6] using an approach analogous to [32,33], which constructs a total union of all regions and advects the “top layer” (see Figure 3b). To perform the advection with the correct surface speed of the underlying material region, it is necessary to detect the active material for each point in the top layer. This active material for a point x is obtained by querying the value of all level-sets at x. The material of the level-set with the smallest value is considered active. Additionally, the level-sets have a fixed order and the lower level-set is chosen as active material, if the values are numerically identical. The etching itself is conducted by advecting the top layer, and subsequently transferring the removal of the material to the underlying level-sets representing the material regions using a Boolean operation between the top layer and each material region. One significant advantage of this top layer approach is that material layers can be represented with sub-grid resolution. This is possible if the level-sets representing the materials are chosen to not map directly to the material regions, but are constructed additively.

**Figure 4:**
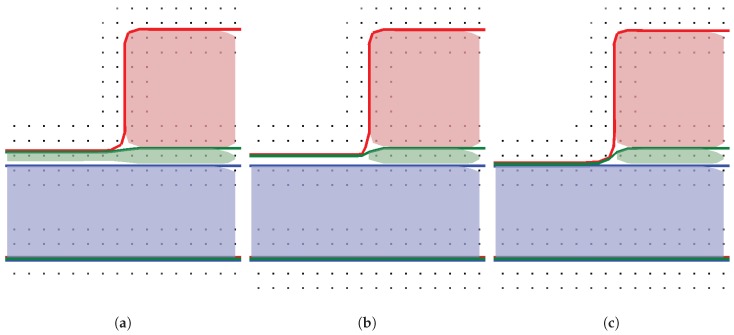
Exemplary development of material stack shown in Figure 3. (**a**) beginning of etching process; (**b**) green layer in sub-grid resolution; (**c**) green layer fully etched.

The chronological development of an exemplary material stack using the top layer advection is shown in Figure 4. Starting from Figure 4a, the green layer is represented with sub-grid resolution (see Figure 4b), until it is fully etched away in Figure 4c. In a time step where a region of a layer is fully etched and the underlying material becomes active, the surface velocities of the involved materials must be averaged. If only the surface velocity for the green layer is considered for the full time step, the surface advection speed is too slow or too fast, if the surface velocity for the blue material is faster or slower, respectively.

### 2.3. Material Interface-Aware Iterative Partitioning

The approach presented in [6] is used to obtain the surface flux rates. The main idea herein is to select a subset (sparse set) of the triangles present in the surface mesh, to minimize the number of integration points used in the flux calculation process. The sparse set is created starting by selecting randomly surface cells with a fixed minimum edge distance of $d_{\max}$, i.e., the minimum number of edges to be crossed to connect two cells on the surface mesh. The iterative partitioning scheme to refine this initial sparse set is guided by two refinement conditions: a threshold for the difference of the normal angles of two neighboring sparse points and a threshold based on the flux difference between two neighboring sparse points. If the normal angle of two neighboring points exceeds the threshold value, the region in between is marked for further refinement. The obtained solution for the sparse set is then extrapolated constantly into the surrounding patch of each sparse point and smoothed using a diffusion approach.

The flux calculation algorithm using the sparse set is comprised of four major parts: (a) the initial partitioning of the surface with a maximum distance $d_{\max}$ between sparse points, (b) the calculation of the flux rates at the sparse points, (c) the refinement of the sparse set where new points are added according to the refinement conditions, and (d) the calculation of the flux rates for the recently added points. Steps (c) and (d) are executed iteratively until a minimum distance $d_{\min}$ between sparse points is reached. The flux calculations steps (b) and (d) are parallelized with OpenMP, whereas the initial partitioning (a) and the refinement (c) are implemented serially.

The refinement condition presented in [6] is used in the multi-material iterative partitioning scheme for the sparse surface evaluation using the maximal normal deviation $\nu_{\max}$, the average flux difference $u_{\mathrm{avg}}$, and the maximum flux difference $u_{\max}$. A combination of thresholds for the normal deviation ($t_{\mathrm{angle}}$) and the flux difference ($t_{\mathrm{flux}}$) is used in all of the following results to model the refinement condition RC for a sparse surface location *i*:

(3) $$RC\left(i\right)=\{\begin{array}{cc} true, & \mathrm{if}\;\nu_{max_{i}}>t_{\mathrm{angle}},\\ true, & \mathrm{if}\;\frac{u_{avg_{i}}+u_{max_{i}}}{2}>t_{\mathrm{flux}},\\ false, & \mathrm{otherwise}. \end{array}$$

$d_{\max}=32$ is used in all simulations, which gives a total of six iterations, whereas the number of Jacobi iterations (for smoothing of the constant extrapolation) is fixed to $d_{\max}/4=8$.

The refinement condition aims at capturing geometric features as well as high gradients in flux distribution. However, different simulation scenarios might require a tailored refinement condition to ensure proper refinement of the sparse set in relevant regions.

**Figure 5:**
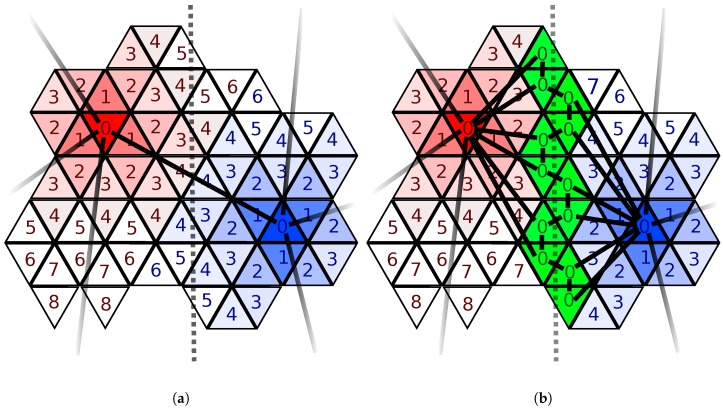
Example region of top layer across a material interface (dotted line). Cells of the sparse set are labeled with 0, while the cells forming the surrounding patch are labeled with their edge distance to the sparse set. Solid black lines denote the connection between the cells of the sparse set. (**a**) the region is partitioned into two patches (red and blue) using $d_{\max}=8$, omitting material information; (**b**) material interface-aware partitioning using $d_{\max}=0$ for cells embedding the material interface: the green cells, which are part of the sparse set, follow the material interface and thus allow for a proper representation.

Since different materials experience potentially high differences in their surface velocities, it is important to ensure a material interface-aware partitioning. Therefore, we extend the scheme presented in [6] by identifying all cells on the top layer embedding a material interface and subsequently set $d_{\max}=0$ for these surface cells. The effect of this material-interface-aware partitioning is shown in Figure 5, where all cells on the interface are present in the sparse set. Thus, we ensure that the sparse set contains a high amount of cells in the material interface regions.

### 2.4. Simulation Setup

From the different process steps in the Dual-Damascene process (see Figure 2), we focus solely on the simulation and evaluation of the etching step indicated in Figure 2g,h. Thus, this section describes the details of the simulation setup of this etching process step.

**Figure 6:**
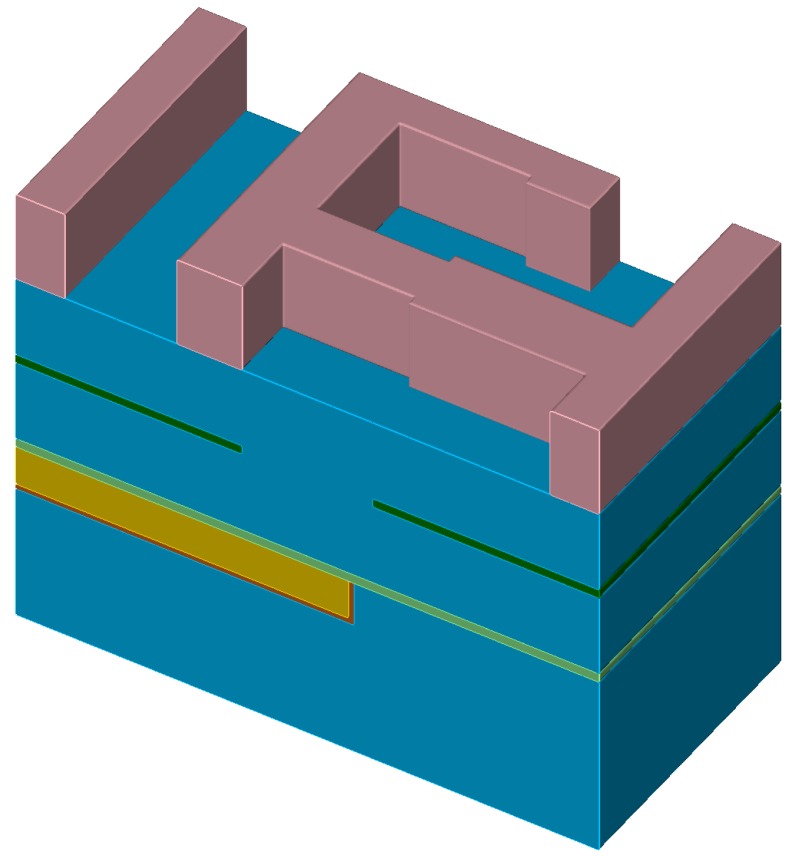
Simulation geometry used in our study (see Figure 2g). Material regions are encoded using colors according to Table 1.

The used normal surface velocity is defined as

(4) $$V_{n}\left(x\right)=\alpha \left(M\left(x\right)\right)\cdot R\left(x\right),$$

where α is denoting a material dependent scalar weighting factor and *R* is the direct flux from the ion source. Figure 6 depicts the simulation geometry used in this study and Table 1 lists the applied scalar weighting factors normalized to the weighting factors of the dielectric.

We set equal weighting factors for the etch stop and the diffusion barrier (α=0.02) as well as for copper and the metal seed layer (α=0.1). The weighting factor of the hard mask is set to α=0.01. These values were arbitrarily chosen to highlight the approach’s ability to handle highly different surface reactions in a multi-material simulation.

A power cosine source with exponent n=1000 [22] is used to model a highly vertically focused ion source, where only the direct flux is considered. In order to compute the direct flux rates on the surface elements, we use the method based on an icosahedron presented in [22], with the subdivision factor set to 5.

Each simulation is conducted until the simulation time T=2.0, where the edge stop layer and the diffusion barrier are reached at T≈0.5 and T≈1.0, respectively. We investigate level-set resolutions ranging from 32 to 128 cells per unit length resulting in 140 to 560 timesteps (Table 2).

The choice of the threshold values must be chosen according to the simulated process and which information will be extracted from the simulation results. To investigate their influence on the accuracy and performance of the flux calculation, we varied the parameters of the refinement conditions around their default values [6]. The threshold $t_{\mathrm{angle}}$ for the difference of the normal angles between two sparse points was set to three different values: 0.05, 0.1, and 0.2. For the flux difference, we set the threshold $t_{\mathrm{flux}}$ to 5, 10, and 20% of the global maximum flux calculated in each timestep.

To assess the influence of the sparse surface evaluation, we perform each simulation twice: once using the sparse surface evaluation (sparse approach) and once using a full evaluation (dense approach)—the latter represents the conventional reference approach.

We investigate the scalability for a fixed problem size (i.e., strong scalability) for both the dense and the sparse approach, with 1, 2, 4, 8, and 16 threads. The execution times of the major parts of the sparse flux calculation (a)–(d) are tracked individually.

A single node of the Vienna Scientific Cluster 3 (VSC-3) [34] is used for all simulations. This node has 64 GB main memory and is comprised of two Intel Xeon E5-2660v2 Sandy Bridge EP processors with 10 physical cores on each processor running at 2.20 GHz. Hence, a total of 20 physical and 40 logical cores are available.

## 3. Results and Discussion

### 3.1. Performance and Accuracy

**Figure 7:**
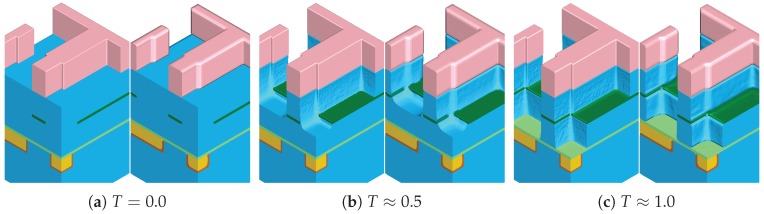
Resulting material regions of the multi-material etching simulation of the dielectric layer for resolution 128 (left half of each figure) and 32 (right half) at different times *T*.

The results at different times *T* during the etching simulation of the dielectric layer when using the sparse surface approach with threshold values $t_{\mathrm{flux}}=10\%$ and $t_{\mathrm{angle}}=0.1$ for the refinement condition (3) are shown in Figure 7. The left and right part of each image in Figure 7 shows the results for level-set resolution of 128 and 32 cells per unit length, respectively. Significant rounding of sharp geometrical features is visible for the lower resolution, which is expected due to the implicit level-set representation. Especially for thin material regions (e.g., the etch stop layer), the influence of the level-set resolution is noticeable.

**Figure 8:**
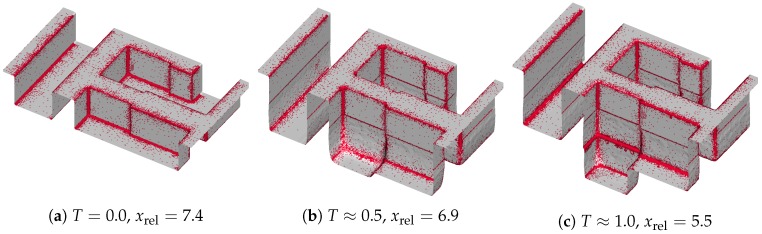
Active cells marked in red for $d_{max}=32$ using resolution 64 at different timesteps.

The sparse sets (colored in red) for resolution 64 at identical times *T* as in Figure 7 are shown in Figure 8. Due to the design of the refinement condition (3), the density of the sparse cells increases towards sharp geometrical features as well as in regions of high flux deviation. Additionally, the material interfaces ($d_{\max}$ is set to 0 at interfaces) are captured within the sparse set. The ratio of the number of cells used in the dense approach to the number of active cells selected with the sparse approach is defined as

(5) $$x_{\mathrm{rel}}=\frac{n_{\mathrm{dense}}}{n_{\mathrm{sparse}}}$$

and ranges from 5.5 to 7.4 in Figure 8.

The factor $x_{\mathrm{rel}}$ denotes the theoretical limit for the obtainable speedup *S* (6), which is the ratio of the flux calculation runtime of the dense approach over the flux calculation runtime of the sparse approach:

(6) $$S=\frac{{\mathrm{time}}_{\mathrm{dense}}^{\mathrm{flux}}}{{\mathrm{time}}_{\mathrm{sparse}}^{\mathrm{flux}}}.$$

To judge the accuracy of the sparse approach, the differences in the surface positions between the sparse and the dense approach are calculated using a fixed timestep interval. A constant Courant–Friedrichs–Lewy (CFL) number of $\alpha_{CFL}=0.4$ is used throughout the simulation [35]. For each surface point, the closest distance (surface deviation) between the two surface meshes is computed.

**Figure 9:**
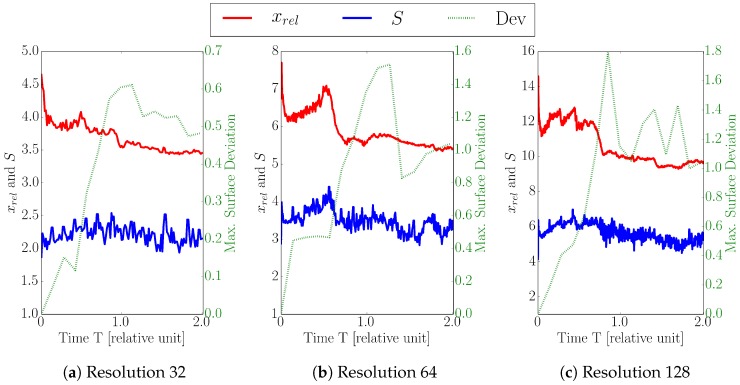
Ratio between dense and sparse number of cells $x_{\mathrm{rel}}$, Speedup *S*, and maximum surface deviation (Dev) for level-set resolution of (**a**) 32, (**b**) 64, and (**c**) 128 using the default values for both refinement conditions ($t_{\mathrm{flux}}=10\%$ and $t_{\mathrm{angle}}=0.1$). The average values are depicted in Figure 10.

For the three different resolutions (32, 64, 128), the relative cell factors $x_{\mathrm{rel}}$, the obtained speedups, and the maximum surface deviations are depicted in Figure 9. For a level-set resolution of 32, we obtain speedups between 1.9 and 2.5 (see Figure 9a), which is about 46% below the theoretical maximum of 4.6. The obtained speedup increases with increasing level-set resolution, which can be seen in Figure 9b,c: for a resolution of 64, we achieve speedups from 2.8 to 4.4, where $x_{\mathrm{rel}}$ reaches at most 7.7. With a resolution of 128, we obtain speedups ranging from 4.1 to 7.0 where $x_{\mathrm{rel}}$ peaks at 14.6. The observed gap between the actual speedup *S* and the theoretical limit ($x_{\mathrm{rel}}$) stems from the computational overhead occurring in the sparse method, which is investigated in detail in Section 3.3. The total runtimes of the dense and the sparse simulations are shown in Table 3 for different resolutions.

Figure 9c shows that the resulting maximum surface deviations are below 1.8 grid cells for the highest resolution (i.e., 128), whereas the maximum deviations for resolution 32 and 64 stay below 0.7 and 1.6 grid cells, respectively. These maximum deviations occur in the vias, where for resolutions 32 and 64 the deviations are about 3% and 1.1% of the size of the via, respectively. For the highest resolution of 128 the maximum deviations correspond to 0.6% of the via size, so the relative maximum deviation decreases with increased resolution. The peak of the maximum surface deviation is for all resolutions around T=1.0, where the etching process reaches the bottom of the via (diffusion barrier), and the high vertical surface velocities occurring in the dielectric are not present anymore. Therefore, in the following timesteps, the maximum surface deviations remain more or less static.

### 3.2. Impact of Variation of Threshold Values

**Figure 10:**
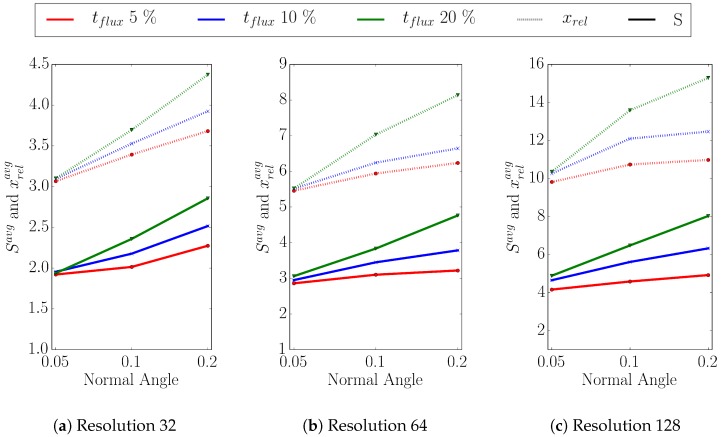
Average speedups $S^{avg}$ (solid lines) and average ratios $x_{\mathrm{rel}}^{avg}$ (dotted lines) for three different flux (red, blue, green) and three different normal angle thresholds (x-axis) for level-set resolutions of (**a**) 32, (**b**) 64, and (**c**) 128.

The sensitivity of the results with respect to the choice of threshold values used in the refinement condition (3) is presented in the following. Figure 10 shows the average speedup and the average $x_{\mathrm{rel}}$ obtained for flux thresholds $t_{\mathrm{flux}}$ of 5, 10, and 20% and normal angle thresholds $t_{\mathrm{angle}}$ of 0.05, 0.1, and 0.2 using three different level-set resolutions (32, 64, and 128).

For simulations with a level-set resolution of 32 (Figure 10a), the ratio $x_{\mathrm{rel}}$ increases with increasing normal angle as well as with increasing flux rate threshold, most prominently for $t_{\mathrm{flux}}=20\%$. The reason for this effect is that the condition if a point has to be added to the sparse set is based on either a violation of the flux rate threshold *or* the normal angle threshold. Therefore, the higher the flux or the normal angle threshold, the smaller the number of points in the sparse set. This also holds for the other two level-set resolutions depicted in Figure 10b,c. As stated before, the discrepancy between the ratios and the achieved speedup shows the computational overhead introduced with the iterative partitioning scheme (Section 3.3).

The influence of the threshold variations on the surface deviations is summarized in Table 4. We categorize the surface cell deviations in bins of size 0.1 ranging from 0.0 to 0.4 and above 0.4. The table contains the percentage of the respective bin population. For all resolutions, an increase in the thresholds leads to an increase in the deviations. The percentage of the surface with a deviation above 0.4 increases from the smallest ($t_{\mathrm{angle}}=0.05$, $t_{\mathrm{flux}}=5$) to the highest ($t_{\mathrm{angle}}=0.2$, $t_{\mathrm{flux}}=20$) threshold configurations, from 0.02 to 0.68% for resolution 32. For resolutions 64 and 128, the percentage of cells having a deviation above 0.4 increases from 0.28 to 3.8% and 0.27 to 5.62%, respectively.

### 3.3. Parallel Scalability

For the scalability investigation of the dense and the sparse approach, we evaluated the flux calculation execution times for both using 1, 2, 4, 8, and 16 threads. The thresholds in the sparse approach were set to ($t_{\mathrm{angle}}=0.1$ and $t_{\mathrm{flux}}=10$).

**Figure 11:**
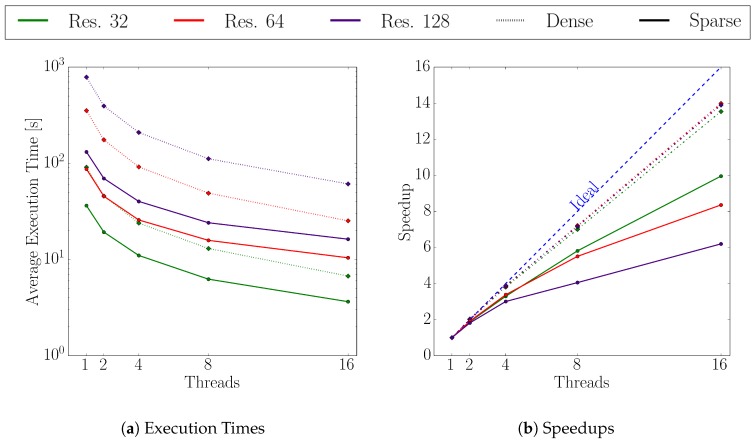
Execution times and speedups at various level-set resolution for the dense and sparse flux calculation approach.

Figure 11 depicts the average execution times and average speedups of the flux calculation using both, the dense (dotted lines) and the sparse (solid lines) approach. The average execution time using the sparse set for the flux calculation is always below the dense set’s time, with the biggest difference occurring for a level-set resolution of 128. Here, the sparse set using one thread and 16 threads is about 7.5 and 3.4 times faster, respectively. The scaling for all resolutions is better with the dense approach where the difference to the sparse set speedup increases for higher resolutions. Using the dense approach with the highest level-set resolution of 128, the resulting speedup with 16 threads is 26% larger than the one from the sparse set. The differences in the execution times, especially for smaller thread counts, originate from the difference in the used number of surface cells for the flux calculation. However, with increasing thread count, the gap between the execution times of the two approaches shrinks, but the sparse method still outperforms the reference approach for 16 threads. The scaling of the sparse set method is better for the lowest resolution of 32, but the sparse speedup for 16 threads is still 26% below the dense speedup. For resolutions 64 and 128, the sparse speedup is about 40% and 55% below the dense’s, respectively.

**Figure 12:**
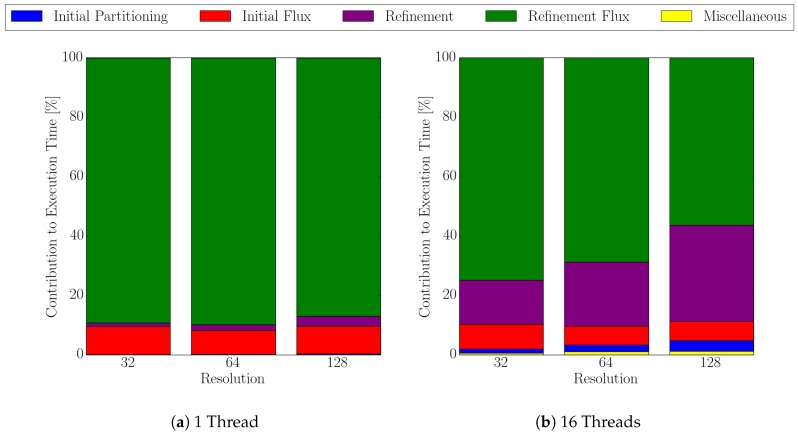
Contribution of the four major steps in the sparse flux calculation algorithm using 1 and 16 threads for different level-set resolutions: initial partitioning of the cells for the sparse set (blue), initial flux calculation (red), iterative refinement (purple), and the corresponding flux calculation (green). Miscellaneous (yellow) accounts for any other occurring overhead within the sparse set algorithm, e.g., computing the local flux deviations from the global maximum flux.

Figure 12 shows the different shares of the overall execution times for 1 to 16 threads for a single timestep. For the serial case (Figure 12a), the time spent for the refinement of the sparse set is only about 3%, whereas the flux calculation for the refined surface cells accounts for about 87%. This relation changes for 16 threads (Figure 12b) where the share for the refinement step increases nearly 10 times to about 32%, and the share of flux calculation for the refined cells decreases to about 56%. The data shown in Figure 12 shows that the serial refinement step for the creation of the sparse set is a bottleneck in our approach. Hence, the limited scalability compared to the dense set approach (cf. Figure 11). Although the scalability within the sparse set is limited, it still significantly outperforms the dense set approach in terms of execution time.

## 4. Conclusions

We have shown that the flux calculation for a multi-material etching simulation of a dielectric layer in the Dual-Damascene process can be accelerated using our recently developed multi-material interface-aware surface evaluation approach. For different surface resolutions and threshold values for the flux difference and the normal angle deviation in our refinement condition for the sparse set, we obtain speedups from 1.9 to 8.0. Our approach introduces minor surface deviations in the surface positions, where the maxima occur inside the vias and are below two grid cells for the highest resolution, corresponding to about 0.6% of the via size. For the lowest resolution, we obtain deviations below 0.7 grid cells which corresponds to 3% of the via size. We evaluated the scalability for a fixed problem size for up to 16 threads, revealing a nearly linear scaling for up to four threads for both the dense and the sparse set approach. Currently, the serial implementation of the iterative partitioning limits the speedup to roughly 50% of the theoretical limit, i.e., the ratio of the size of the dense and sparse set. However, our sparse approach still massively outperforms the conventional approach in terms of execution time. We plan to overcome this limitation by adopting the partitioning scheme to allow for a parallel implementation.

To increase the general applicability of the presented approach, we also aim to support reemitted flux. Either the already existing sparse set is reused and further refined where necessary or a dedicated sparse set is created in each subsequent reemission step. Additionally, it could be an advantage to adapt the refinement condition for the calculation of the reemitted flux distributions.

References

## Figures and Tables

**Table 1:** Normalized weighting factors corresponding to the etch rates for the different materials in our simulation.

	Material M	Weighting Factor α
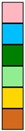	Hard Mask	0.01
	Dielectric	1.0
	Etch Stop	0.02
	Diffusion Barrier	0.02
	Copper	0.1
	Metal Seed	0.1

**Table 2:** Investigated level-set resolutions, corresponding initial domain resolutions, the number of triangles and vertices in the initial surface mesh, and the number of required timesteps until simulation time T=2.0.

Cells per Unit Length	Vertical Cells	Horizontal Cells	Triangles	Vertices	Timesteps
32	80	96 × 96	28,642	56,384	140
64	160	192 × 192	113,986	226,176	280
128	320	384 × 384	455,298	907,008	560

**Table 3:** Total simulation runtimes for different level-set resolutions using the dense and sparse approach.

Resolution	Runtime Dense [min]	Runtime Sparse [min]
32	22	12
64	159	60
128	1209	311

**Table 4:** Distribution of surface deviations (normalized to the cell size) at T≈1.0 for different level-set resolutions, normal angle, and flux thresholds. The population of the bins is given in percent.

Resolution 32										
	**Angle**	**0.05**			**0.1**			**0.2**		
	**Flux**	**5**	**10**	**20**	**5**	**10**	**20**	**5**	**10**	**20**
**Deviation**	0.0–0.1	98.62	98.05	98.45	94.40	93.66	89.67	91.12	86.24	81.33
	0.1–0.2	1.17	1.57	1.26	4.31	4.97	8.65	5.55	7.06	10.82
	0.2–0.3	0.15	0.22	0.18	1.10	1.27	1.49	2.37	3.37	4.84
	0.3–0.4	0.04	0.10	0.07	0.13	0.09	0.11	0.87	2.74	2.33
	>0.4	0.02	0.06	0.04	0.07	0.01	0.08	0.08	0.58	0.68
**Resolution 64**										
	**Angle**	**0.05**			**0.1**			**0.2**		
	**Flux**	**5**	**10**	**20**	**5**	**10**	**20**	**5**	**10**	**20**
**Deviation**	0.0–0.1	96.42	92.91	92.55	96.10	89.69	85.26	95.73	88.42	80.42
	0.1–0.2	2.63	5.84	6.05	2.93	7.99	8.52	3.31	9.01	9.88
	0.2–0.3	0.46	0.71	0.80	0.51	1.67	3.14	0.51	1.88	3.76
	0.3–0.4	0.22	0.25	0.28	0.18	0.36	1.89	0.17	0.37	2.13
	>0.4	0.28	0.29	0.32	0.27	0.30	1.19	0.27	0.31	3.80
**Resolution 128**										
	**Angle**	**0.05**			**0.1**			**0.2**		
	**Flux**	**5**	**10**	**20**	**5**	**10**	**20**	**5**	**10**	**20**
**Deviation**	0.0–0.1	88.11	84.26	84.26	86.08	80.20	76.90	85.46	78.55	73.65
	0.1–0.2	8.92	9.89	9.59	10.35	11.60	11.69	10.68	12.24	12.11
	0.2–0.3	2.19	3.66	3.54	2.56	4.56	4.92	2.70	5.16	5.77
	0.3–0.4	0.50	1.38	1.57	0.68	2.13	2.26	0.76	2.26	2.86
	>0.4	0.27	0.81	1.04	0.32	1.51	4.23	0.40	1.79	5.62
